# Epidemiology of Moderate Alcohol Consumption and Breast Cancer: Association or Causation?

**DOI:** 10.3390/cancers10100349

**Published:** 2018-09-22

**Authors:** Samir Zakhari, Jan B. Hoek

**Affiliations:** 1 Science Office, Distilled Spirits Council, Washington, DC 20005, USA; 2 Department of Pathology, Anatomy and Cell Biology, Thomas Jefferson University, Philadelphia, PA 19107, USA

**Keywords:** alcohol, breast cancer, epidemiology, risk factors, genetics, epigenetics, molecular pathological epidemiology, meta-analysis, moderate drinking

## Abstract

Epidemiological studies have been used to show associations between modifiable lifestyle habits and the incidence of breast cancer. Among such factors, a history of alcohol use has been reported in multiple studies and meta-analyses over the past decades. However, associative epidemiological studies that were interpreted as evidence that even moderate alcohol consumption increases breast cancer incidence have been controversial. In this review, we consider the literature on the relationship between moderate or heavy alcohol use, both in possible biological mechanisms and in variations in susceptibility due to genetic or epigenetic factors. We argue that there is a need to incorporate additional approaches to move beyond the associations that are reported in traditional epidemiological analyses and incorporate information on molecular pathologic signatures as a requirement to posit causal inferences. In particular, we point to the efforts of the transdisciplinary field of molecular pathological epidemiology (MPE) to evaluate possible causal relationships, if any, of alcohol consumption and breast cancer. A wider application of the principles of MPE to this field would constitute a giant step that could enhance our understanding of breast cancer and multiple modifiable risk factors, a step that would be particularly suited to the era of “personalized medicine”.

## 1. Introduction

Breast cancer presents as solid tumors that originate in breast tissue due to deleterious mutations in different cell types, most commonly originating in ductal epithelium. As is true for most cancers, there are multiple subtypes, which have different etiologies and require different treatments. Breast cancer remains a global concern notwithstanding the 2015 National Cancer Institute fact sheet [1], stating that overall breast cancer death rates continued to decrease in the United States in the period between 1990 and 2014 (from 33.1 to 20.5 per 100,000 women). Breast cancer represents the second most common female malignancy worldwide and is one of the primary causes of death among women globally [2].

All cancers are subject to a combination of intrinsic and accidental factors that reflect the balance of the impact of inborn as well as incidental deleterious mutations and the intrinsic cellular and tissue repair and other defense mechanisms available in an individual. A vast literature exists aimed at differentiating between such intrinsic and modifiable risk factors. Epidemiological studies have been used extensively to address these questions.

In this review, we focus in particular on epidemiological studies that have attempted to assess the role of alcohol consumption as a risk factor in the development of breast cancer. Epidemiology applies statistical approaches to identify associative relations; such studies by themselves are intrinsically less suitable to identify causal relationships: Establishing causality requires additional information. Furthermore, apart from complications due to confounding factors that may be difficult to assess, the validity of the association depends critically on the quality of the variables of interest, in this case, the reliability of the information on respondents’ alcohol consumption, both in quantities consumed and drinking profile, throughout an individual’s history relevant to disease onset and progression. Alcohol use history is almost invariably estimated based on self-reporting and averaged over shorter or longer time periods. This is particularly problematic when investigating the association of more moderate alcohol use with breast cancer incidence. Our goal is to provide a more detailed evaluation of the arguments in support of the validity of the suggested causality between alcohol drinking, and in particular moderate drinking, and the incidence and progression of breast cancer. We will consider possible mechanisms that could underlie such a relationship and emphasize the need for supplementary evidence, be it biological plausibility or pathological supportive information, that could help strengthen the scientific basis on which conclusions are based.

### 1.1. Molecular Characteristics of Breast Cancer Subtypes

Breast cancer is a heterogeneous disease that was classified by the World Health Organization (WHO) [3] to encompass 21 distinct histological types. Roughly 95% of invasive breast cancers are adenocarcinomas that originate in the epithelial tissue of the terminal duct lobular unit [4]. DNA microarray and next-generation sequencing technology has been used to characterize gene expression in breast cancer and identify its molecular subtypes. In addition, immunohistochemical stains are routinely used to identify the status of hormone receptors: estrogen receptor (ER), progesterone receptor (PR), and human epidermal growth factor receptor 2 (HER2). The molecular and clinical heterogeneity shown by specific gene expression signatures can predict mortality risk, metastasis and survival [5]. 

Breast cancers have been classified [6] into five major subtypes. (1) luminal A (ER and/or PR-positive, HER2-negative); (2) luminal B (ER-positive and/or PR-positive, HER2-positive); (3) *HER2 overexpressing* (ER-negative, PR-negative, HER2-positive); (4) *basal-like* (ER-negative, PR-negative, HER2-negative, cytokeratin 5/6-positive, and/or HER2-positive); and (5) unclass­ified breast tumors [7]. Tumors in subtypes four and five have a triple-negative (TN) phenotype (ER-negative, PR-negative, HER2-negative); however, approximately 70% of TN tumors are basal-like [8]. Risk factors associated with ER-positive and PR-positive breast tumors involve mechanisms related to endogenous hormones, whereas the genesis of ER-negative and PR-negative breast cancers may be non-hormonal [9].

The cell origin of breast cancer has been debated. Two models (not mutually exclusive) have been advanced: The sporadic clonal evolution model (stochastic), which suggests that any epithelial cell can undergo random mutations that compete for survival and progression; and the *cancer stem cell model* (hierarchical) which postulates that stem cells give rise to the tumor or were acquired within the tumor and, due to their self-renewal ability, maintain tumorigenesis [10].

### 1.2. Risk Factors for Breast Cancer

Breast cancer is a multifactorial disease resulting from a series of interactions between genetics, environmental factors, immune response, and even breast microbiota. A multitude of factors, both modifiable and non-modifiable, can contribute to the risk of breast cancer. While some mutations can be inherited at birth, the majority are acquired later in life due to various risk factors.

The strongest non-modifiable primary risk factors for breast cancer include aging; reproductive parameters which determine the cumulative lifetime estrogen exposure [early menarche (before age 12); delayed menopause (after age 55); delayed childbearing (first full-term pregnancy after age 30); miscarriage; abortion]; and genetics (inherited changes in certain genes and family history of breast cancer) [11].

Various modifiable lifestyle risk factors have been identified, including dietary habits (consumption of polyunsaturated fats and excessive alcohol), smoking, exposure to radiation or synthetic estrogens, physical inactivity, use of hormone-replacement therapy (HRT) or diethylstilbestrol, obesity, diabetes, breast implants, epigenetic factors, viruses (e.g., Epstein-Barr virus, or EBV; human papillomavirus, or HPV), occupational exposure to chemicals (e.g., polychlorinated biphenyls, or PCBs; solvents; and exposure to endocrine-active chemicals that can disrupt typical endocrine behaviors), exposure to pesticides and air pollution, and even night-shift work. Needless to say, other risk factors may exist that have not yet been fully identified. For example, a higher relative abundance of bacteria that have the ability to cause DNA damage, and a decrease in some anticarcinogenic lactic acid bacteria were detected in breast cancer patients, which raises the question of the role of the mammary microbiome in modulating the risk of breast cancer development [12].

The risk attributable to modifiable and non-modifiable factors was calculated in a German population-based, case-control study of postmenopausal breast cancer [13]. The attributable risk for non-modifiable risk factors was 37.2% regarding overall invasive tumors; 36.5% for ER+/PR+ tumors; 47.9% for ER+/PR− tumors; and 31.1% for ER−/PR− tumors. Of the modifiable risk factors, hormone replacement therapy and physical inactivity had the highest impact on breast cancer-attributable risk (19.4% and 12.8% respectively for overall invasive tumors; 25.3% and 16.6%, respectively, for ER+/PR+ tumors). Odds ratios for alcohol consumption and body mass index (BMI) were not statistically significant regarding invasive breast cancer risk. Different risk factors were associated with specific types of breast cancer; for example, obesity was associated with increased risk of the TN subtype, while older age and use of hormone replacement therapy was associated with increased risk of HER2 overexpressing tumors [6].

In the following sections we will briefly discuss genetic and epigenetic risk factors to highlight the complexity of breast cancer etiology and to point to the impact of alcohol use as a modifiable risk factor in specific genetic and epigenetic contexts. A better understanding of such gene-environment interactions may help identify potential biological pathways and shed light on breast cancer etiology.

#### 1.2.1. Genetic Factors

The incidence of breast cancer in first-degree relatives of women with the disease is about twice that of the general population [14]. Thus, the disease exhibits familial aggregation. However, the majority of breast cancers are not hereditary and occur in the absence of a first-degree family history of breast cancer.

Breast cancer susceptibility genes can be categorized as high-, moderate-, or low-penetrance based on the relative risk of breast cancer associated with these genes’ mutations. These susceptibility genes were discussed by Apostolou and Fostira in 2013 [15], and 84 independent loci have been identified by Genome-wide association studies (GWAS) [16].

High-penetrance genes, such as *BRCA1*, *BRCA2*, p53, *PTEN*, *STK11*, and *CDH1* are associated with a relative risk of breast cancer development higher than 5.

Mutations in two prominent susceptibility genes, *BRCA1* and *BRCA2*, were identified as major risk factors for breast cancer [17,18]. However, the incomplete penetrance of these mutations suggests that other factors—environmental, hormonal, or modifier genes—may modify that risk. This is illustrated by a study in which monozygotic (MZ) twins who carried identical *BRCA1* gene mutations, resulted in discordant phenotypes: One had breast cancer twice in 27 years while her MZ twin remained healthy [19]. *BRCA1*-associated cancers are more likely to present specific characteristics like the absence of ER, PR, and HER2 receptors [20,21], unlike *BRCA2*-related cancers, which usually express ER and PR. Examination of the role of BRCA1 and BRCA2 in the DNA damage response has led to the identification of several breast cancer susceptibility genes such as *FALB2*, *CHEK2*, *BRIP1*, all of which interact directly or indirectly with BRCA1 or BRCA2 [22]. Other studies reported that alcohol consumption does not appear to increase breast cancer risk in premenopausal young women carrying a *BRCA* gene mutation [23,24].

Moderate-penetrance genes confer a relative increase in the risk of breast cancer between 1.5 and 5 [25]. GWAS have identified genetic susceptibility variants of medium-penetrance which confer an increased risk per allele of 2 to 3. These include variants in *PALB2* (which encodes a protein colocalizing with BRCA2) [26]; checkpoint kinase 2 (*CHEK2*) [27]; ataxia telangiectasia mutated (*ATM*) [28]; and *BRCA1*-interacting protein C-terminal helicase 1 (*BRIP1*) [29]. Studies on TN breast cancer patients showed associations of breast cancer with mutations in moderate penetrance breast cancer susceptibility genes *FALB2*, *BARD1*, *BRIP1*, *RAD51C*, and *RAD51D* [30].

Low-penetrance genes are associated with a relative increase in the risk of breast cancer of about 1.5. Four loci were identified that exhibited consistent association with breast cancer and contain plausible causative genes [31]: fibroblast growth factor receptor 2 (*FGFR2*)*;* TOX high mobility group box family member 3 (*TNRC9*)*;* mitogen-activated protein kinase kinase kinase 1 (*MAP3K1*)*;* and lymphocyte-specific protein 1 (*LSP1*).

The susceptibility to breast cancer is polygenic; i.e., conferred by a large number of loci, each with limited contribution to breast cancer risk [32]. Some of the low-penetrance alleles are more specific to ER-negative or TN tumors, particularly in younger women (less than 50 years of age). These include telomerase reverse transcriptase (*TERT*) [33] and ethylene response sensor 1 (*ERS1*) [34]; while others (e.g., *FGFR2*) are more specific for ER-positive breast cancer [35]. 

Additional susceptibility loci have been identified for breast cancer in several recent studies. For example, a 2014 study showed association between breast cancer risk and seven breast cancer susceptibility loci (*FGFR2*, *TOX3*, *STXBP4*, *SLC4A7*, *LSP1*, 2q35, and 5p12) [36]. A genome-wide association study identified 25 known breast cancer susceptibility loci as risk factors for TN breast cancer. Another study confirmed an association with TN breast cancer for 10 loci (*LGR6*, *MDM4*, *CASP8*, 2q35, 2p24.1, *TERT*-rs10069690, *ESR1*, *TOX3*, 19p13.1, *RALY*), and identified 15 additional breast cancer loci (*PEX14*, 2q24.1, 2q31.1, *ADAM29*, *EBF1*, *TCF7L2*, 11q13.1, 11q24.3, 12p13.1, *PTHLH*, *NTN4*, 12q24, *BRCA2*, *RAD51L1*-rs2588809, and *MKL1*) [37]. In addition, a 2013 meta-analysis of nine genome-wide association studies (including 10,052 breast cancer cases of European ancestry) identified 41 new loci associated with breast cancer risk [38]. A 2014 genome-wide association analysis of more than 120,000 individuals identified 15 new susceptibility loci for breast cancer [39]. By using methods accounting for gene-environment interaction, researchers identified three single-nucleotide polymorphisms, or SNPs (rs12197388 on chromosome 6; rs10483028 and rs2242714 on chromosome 21), showing a statistically significant association between 10 environmental variables and risk for breast cancer [40].

Other risk factors reported for breast cancer include mutations in the p53-inducible protein phosphatase *PPM1D* (protein phosphatase, Mg^2+^/Mn^2+^ dependent, 1D) [41], *MTHFR* (methylene­tetrahydrofolate reductase), Ala222Val gene polymorphisms [42], and *APOBEC3B* (apolipoprotein B mRNA editing enzyme, catalytic polypeptide-like 3B) overexpression [43].

Studies [44,45] conducted about a decade ago showed that the D302H polymorphism in *CASP8* (caspase 8, rs1045485) could reduce breast cancer risk. Further studies confirmed this protective effect and showed that the per-allele odds ratio is 0.88 for the *CASP8* D302H (rs1045485) [46]. 

The above discussion demonstrates the critical role of mutations in tumor suppressors and oncogenes in the nuclear genome (e.g., *BRCA1*, *BRCA2*, p53, *PTEN*, *ATM*, *CHEK2*, *HER2*) in breast carcinogenesis.

Compared to nuclear DNA (nDNA), mitochondrial DNA (mtDNA) is more susceptible to mutations due to limited repair mechanisms. In breast cancer, mitochondrial function is severely impaired [47]. One early event in breast carcinogenesis can be mutations in mtDNA that destabilize the oxidative phosphorylation system (OXPHOS). In addition, various pro- and anti-apoptotic family proteins regulate glycolysis and respiration [48], and OXPHOS defects could lead to suppression of apoptosis or enhancement of mitophagy in breast cancer cells. Numerous alterations in mtDNA in breast cancer have been reported, including point mutations, mtDNA depletion, microsatellite instability, and insertions [49,50,51,52,53]. SNPs in mtDNA, including G9055A, T239C, C16207T, T16519C, and A263G [54], were found to increase breast cancer risk, while others (T3197C, G13708A) were found to decrease breast cancer risk [55].

The incidence of invasive breast cancer or its precursor lesion, ductal carcinoma in situ (DCIS), increases at an exponential rate until menopause, followed by a slower rate of increase, supporting the notion that breast cancer biology is age dependent. Early-onset breast cancer, therefore, could largely represent inherited mutations (*BRCA1*, *BRCA2*, p53, *ATM*, or *PTEN*) or early, life-transforming events that affect the immature mammary cells, whereas later-onset breast cancer may be associated with environmental or lifestyle factors [56]. While susceptibility loci discussed above are by no means an exhaustive list, it shows the polygenic nature of breast cancer and the complexity of various genes (and environment) interactions in breast carcinogenesis.

To what extent these susceptibility loci influence the impact of alcohol consumption on the actual breast cancer development has received scant attention. Several factors have to work in concert to affect alcohol toxicity. For example, a recent study in a mouse model of hematopoietic stem cells reported that the Fanconi anemia DNA-repair pathway counteracts the genotoxic effects of acetaldehyde produced by alcohol metabolism. Only animals with combined inactivation of aldehyde catabolism (through Aldh2 knockout—ALDH2, the mitochondrial aldehyde dehydrogenase that is primarily responsible for oxidation of acetaldehyde) and the Fanconi anemia DNA-repair pathway (Fancd2 knockout) display susceptibility to the toxic effects of ethanol; the Fanconi anemia pathway prevents aldehyde lesions from degenerating into DSBs [57]. A critical role for acetaldehyde and oxidative stress damage in alcohol-associated liver and esophageal cancer has been described in the studies by Seitz and coworkers [58] and others. Similarly, alcohol-induced damage to mitochondrial function has been well established, particularly at higher levels of alcohol use [59,60]. These studies also point to the requirement for significant acetaldehyde accumulation before any of these effects of alcohol exposure can be detected. For instance, extensive studies on the health consequences of alcohol use on individuals carrying a defective copy of ALDH2, primarily from East Asian counties, has highlighted the increased risk for esophageal cancers [61]. Thus, these studies support the interpretation that alcohol enhancement of DNA damage and interference with mitochondrial quality control are detectable only at relatively high levels of alcohol consumption. To what extent lower levels of acetaldehyde can exert damaging effects over a prolonged period of exposure remains insufficiently established.

#### 1.2.2. Epigenetic Modifications

As described above, mutations in oncogenes and tumor-suppressor genes, and chromosomal abnormalities result in specific gene expression profiles that are characteristic of distinct types of breast cancer and their responses to treatment. These genes are involved in the regulation of cellular homeostasis, including cell proliferation, DNA repair, and survival. However, mammary stem cells undergoing differentiation to primitive progenitor cells that give rise to luminal and myoepithelial progenitors, which in turn differentiate into epithelial subtypes, are under epigenetic control. Epigenetic mechanisms, which result in changes in gene expression patterns without altering DNA sequence, contribute to the mammary gland’s developmental phases from fetal to menopause, as well as in breast carcinogenesis, by changing gene expression patterns for cell differentiation, proliferation and apoptosis. Two major classes of epigenetic modulation include DNA methylation and covalent modifications of histones that determine DNA accessibility. In addition, a multitude of additional mechanisms exist that determine the fate of mRNA, e.g., through differential splicing, or by altering mRNA stability or translation, including through effects of non-coding RNAs such as microRNA (miRNA) and various long non-coding RNAs (lncRNAs), as well as other mRNA modification mechanism [62]. Below, we will briefly discuss these epigenetic mechanisms, as potential mechanisms by which alcohol use may induce changes in the gene expression profiles associated with tumorigenesis.

**Figure 1:**
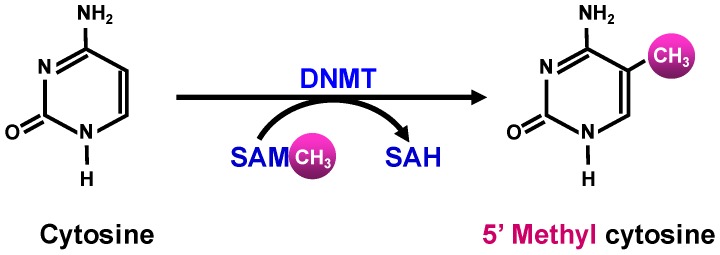
S-adenosyl methionine (SAM) donates a methyl group to cytosine to form 5′ methyl cytosine and S-adenosyl homocysteine (SAH).

DNA methylation involves the transfer of a methyl group from the global methyl donor S-adenosyl methionine (SAM)—predominantly by a set of core DNA methyltransferases (DNMTs)—onto the 5′-position of the cytosine residue in the CpG islands (Figure 1).

**Figure 2:**
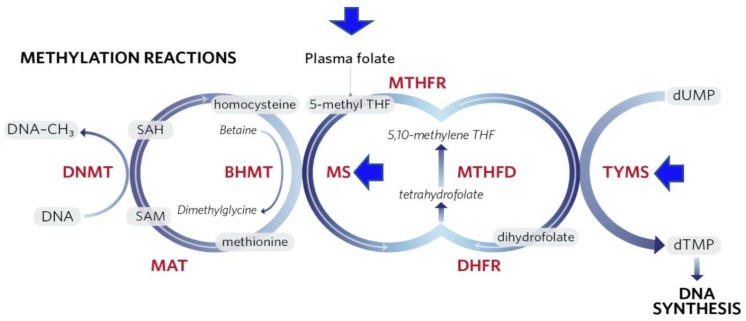
Folate acts as a shuttle for methyl groups that will be used in the metabolism of s-adenosyl methionine (SAM), in de novo synthesis of purines and thymidylate. Dihydrofolate reductase (DHFR) is an enzyme that reduces dihydrofolate to tetrahydrofolate. Arrows point to potential target sites for alcohol-induced changes in methionine cycle function. BHMT = betaine homocysteine methyl transferase; MTHFD = methylenetetrahydrofolate dehydrogenase; DNMT = DNA methyl transferase; MTHFR = methylenetetrahydrofolate reductase; MAT = methionine adenosyltransferase; SAH = S-adenosyl homocysteine; MS = methionine synthase; TYMS = thymidylate synthetase.

SAM is generated in the methionine cycle by condensing ATP with methionine, which itself is generated by re-methylation of homocysteine (Figure 2). This process involves the transfer of a methyl group to homocysteine either from N5-methylene tetrahydrofolate (THF) by methionine synthase, or from betaine, by betaine homocysteine methyl transferase (BHMT) [63]. 

The CpG islands, which exist in about 70% of gene promoters, are generally unmethylated [64]; their methylation results in transcriptional silencing. Importantly, there is considerable literature suggesting that changes in CpG methylation affect the expression and regulation of cancer-related genes. CpG methylation in promoter regions of tumor-suppressor genes (e.g., *BRCA1*, *VHL* and p16Ink4a) leads to the inactivation of these cancer-preventing proteins. Hypermethylation of numerous genes, whose biological functions include *hormone regulation* (ERα, ERβ and PR); *DNA repair* (*BRCA1*, *MGMT*, *MLH1*, and *GST3*); *cell cycle regulation* (p16Ink4a, cyclin *D2*, p14ARF, p57KIP2); *apoptosis* (*DAPK1*, *HOXA5*, *TMS1*, *TWIST*, *FHIT*, *GPC3*); *cell-growth inhibition* (*RARβ*, *TGFβRII*, *SOCS1*, *RASSF1A*, *HIN1*, *NES1*, *SYK*, *WIF1*); *angiogenesis* (maspin and *THBS1*); *invasion* (*TIMP3*, E-cadherin); and *metastasis* (*APC*, *TIMP3*), has been identified in breast tumors [65].

Furthermore, DNA hypermethylation results in aberrant regulation of the Wnt pathway in breast cancer [66], and *BRCA1* expression is suppressed by a combination of gene mutation and DNA hypermethylation [67]. ERα is encoded by *ESR1*, the promoter of which shows a higher degree of methylation in ER^−^ vs. ER^+^ tumors [68]. This hypermethylation of the promoter region of the *ESR1* gene results in a lack of expression of ER, which is reversed by 5-aza-2′deoxycytidine, a DNMT inhibitor [69]. 

DNA hypomethylation can also be a factor in breast carcinogenesis [70]. For example, hypomethylation of repeat elements (such as Alu, α satellites, and *LINE*) promotes chromosomal translocation, deletion, duplication, and genomic instability [71]. This epigenetic change correlates with the tumor size, stage, and histological grade of breast cancer [72]. In addition, promoter hypomethylation could reactivate proto-oncogenes (such as synuclein γ, *ID4*, urokinase, N-cadherin, β-catenin, and annexin A4), which are associated with tumor metastasis and drug resistance to endocrine therapy [73]. 

There is large literature indicating that alcohol consumption can affect the DNA methylation state, both in analyses on human patients and in studies on experimental animals. Some of these changes may be associated with a suppression of the availability of the methyl donor SAM through effects on the methionine cycle (Figure 2). For instance, chronic alcohol feeding to rats for 9 weeks resulted in a decrease in hepatic concentrations of SAM and methionine, as well as 40% reduction in DNA methylation [74]. Christensen and colleagues [75] showed that increasing alcohol intake had a strong trend toward decreased DNA methylation. However, other studies have shown that alcohol consumption was associated with differential alterations in methylation patterns for several genes, e.g., hypermethylation of the ER-α [76] and E-cadherin genes and hypomethylation of p16 [77]. 

Chronic heavy alcohol use can also affect the availability of the folate derivative THF as a methyl donor for methionine synthesis. There is considerable evidence that chronic alcohol use can impair folate uptake and cause folate deficiency, which is likely to affect folate methylation pathways [78]. Thus, low folate levels could promote carcinogenesis by influencing the extent of DNA methylation [79,80] and thereby affect gene expression, DNA integrity and stability [81]. Another way by which low folate levels may mediate carcinogenesis is by hampering the conversion of dUMP to dTMP (Figure 2). Inadequate dTMP levels result in nucleotide deficiency, culminating in DNA strand breaks due to inappropriate incorporation of uracil into DNA in place of thymidine [82]. Furthermore, low dietary folate intake might be associated with breast cancer by hypomethylation of the ER receptor, which may influence silencing genes [74]. Other components that may work in concert with folate, such as Vitamin B12 and methionine, may affect carcinogenesis due to their critical roles in the one-carbon metabolism pathway.

In addition, there is evidence that a woman’s genotype for the MTHFR variant modulates the effect of alcohol consumption on breast cancer risk. For example, women with the TT genotype are at a higher risk of breast cancer than those with other genotypes. In postmenopausal women, the breast cancer risk was increased in women with the C677T MTHFR variant who had high lifetime daily alcohol intake, suggesting that folate metabolism has an impact on cancer development [83]. Despite these arguments, a 2014 meta-analysis [84] of 15 prospective cohort studies and one nested case-control study, including 1,854,013 participants and 24,620 breast cancer patients, showed that dietary folate intake was not significantly associated with the risk of breast cancer. Alcohol stratification analysis was conducted based on six prospective studies, and the results indicated no significant association between high or low dietary folate intake and breast cancer risk. Three of these studies found marked reductions in breast cancer risk among those who consumed greater amounts of alcohol, while the other three studies reported that the relationship between folate intake and breast cancer was not modified by alcohol intake. Furthermore, a 2007 meta-analysis [85] that included only two prospective studies indicated that high folate intake was associated with a statistically significant decreased risk of breast cancer among women with moderate or high alcohol consumption. Thus, the role of methyl donor availability in clinical outcomes mediated by DNA methylation remains a subject for further study.

Histone modification represents another epigenetic mechanism that influences chromatin remodeling during stem and/or progenitor cell differentiation. The histone “tails” that extend from the nucleosome contain lysine residues with ε-amino groups that invariably undergo post-translational modifications, which mainly include methylation, acetylation, phosphorylation, and ubiquitination. Histone tails can enhance or inhibit the accessibility of transcription factors to target loci. Histone acetylation by acetyl transferases (HATs); deacetylation by deacetylases (HDACs); methylation by methyltransferases (HMTs); or demethylation (by histone demethylases; HDMs) further modify chromatin structure. Acetylation of lysine residues in histones results in an open chromatin structure (euchromatin) which allows gene transcription. On the other hand, deacetylation of these lysine residues results in the formation of closed chromatin (heterochromatin) and repression of gene transcription [86]. Methylation of lysine residues results in either activation or repression of chromatin structural conformation. Modifications of H4 generally result in the loss of acetylated K16 and tri-methylated K20 forms, culminating in DNA hypomethylation in human tumor cells, including breast cancer [87]. In addition, numerous non-histone proteins have been reported to be substrates for HDACs. These proteins are involved in transcription (p53, STAT1, STAT3, HMGB, and NFκB), DNA repair (Wnt signaling, β-catenin), heat shock response (HSP90), and hormone response (ERα) [88].

As is true for DNA methylation, histone modification reactions also depend on the availability of an adequate supply of methyl donors or acetyl donors. Interference with the operation of the methionine cycle and the supply of folate and THF could equally limit the rate of histone methylation at a global level. Similarly, the supply of acetyl CoA for histone acetylation may be altered by the availability of this metabolic intermediate. Interestingly, although acetyl CoA is normally derived predominantly from mitochondrial metabolism, which can limit its availability for nuclear acetylation reactions, alcohol metabolism generates abundant acetate as a metabolic product that is largely released from the liver to enter the circulation. Other tissues have the capacity to take up acetate and activate it to acetyl CoA through the cytosolic/nuclear acetyl CoA synthetase-2 (ACSS2). This makes acetyl CoA available systemically during ethanol metabolism and potentially influences histone (or other protein) acetylation in multiple tissues [11,89]. To what extent these processes influence tumorigenesis or tumor progression remains poorly characterized. 

Alcohol use, or other environmental factors can also alter the activity and/or expression of the multiple classes of histone-modifying proteins and their associated factors. As an example, persistent exposure of mammary gland stem and progenitor cells to different environmental factors such as xenoestrogens (phthalates, ethinyl estradiol, phytoestrogens) alters their epigenetic reprogramming during epithelial differentiation [90]. This is mediated, in part, through ERα nuclear receptors that activate or silence the transcription of target genes [91]. Interactions between ERα and various enzymes involved in histone modifications (HAT, HDAC, HMT, HDM), co-activators and co-repressors have introduced another layer of complexity in the epigenetic regulation of breast carcinogenesis [92]. Moreover, several HDACs (HDAC1, 6 and 8) have been found to be upregulated or overexpressed in breast cancers and to interact with histone-lysine-specific demethylase 1 (LSD1) and EZH2, a component of the polycomb repressor complex, playing an important role in the transcriptional changes involved in breast cancer carcinogenesis.

Epigenetic mechanisms acting through mRNA modifications: There is substantial emerging evidence for an impact of alcohol intake on epigenetic regulation through modification of mRNA through splicing factors or by the impact of microRNAs (miRNAs), lncRNAs, or other non-coding RNAs or RNA binding proteins that affect mRNA stability and structure [93]. Like protein-coding genes, the expression of non-coding RNAs and other RNA modifying factors are themselves under epigenetic control by histone modifications and DNA methylation [94].

There has been considerable evidence that miRNAs and other non-coding RNAs play important roles in epigenetic regulation relevant to cancer [95]. Differential expression of miRNAs has been reported during mammary gland development and a miRNA signature for progenitor cells has been identified in mice [96]. miRNAs directly influence stem cell function (miRNA200c) [97] and cell proliferation by regulating cyclooxygenase-2 (miRNA101a) [98] and PTEN (miRNA-205) [99] expression, and up-regulating E-cadherin (miRNA-373) [100]. Most miRNAs that regulate tumor behavior and progression are dysregulated during breast cancer progression. Several miRNAs, like miRNA-206, miRNA17-5p, miRNA-125a, miRNA-125b, miRNA-200, and miRNA-34 and 31, which function as tumor suppressor genes, were lost in breast tumors. Expression of miRNA that were reported to be deregulated and are differentially expressed in normal and breast cancer tissue include downregulation of miRNA-10b, miRNA-125b, and miRNA-145, and upregulation of miRNA-21 and miRNA-155 [101]. Furthermore, epigenetically deregulated microRNA-375 has been shown to be involved in a positive feedback loop with ERα in breast cancer cells [102]. There is an emerging literature pointing to the complexity of the regulatory consequences of all aspects of the tumorigenesis through these mechanisms. However, it is too early to make confident predictions regarding the implications of alcohol-induced deregulation of these processes and their health consequences.

Unlike genetic mutations, epigenetic alterations are potentially reversible under the right circumstances, and therefore are targets for medications development for cancer treatment. Effective treatment for cancer includes the use of DNMT inhibitors and HDAC inhibitors [103]. However, these are blunt tools that do not allow for the selective targeting of the regulation of individual genes or DNA regions.

Similar to other types of cancer, breast cancer initiates and further progresses at the molecular level when specific hallmarks of cancer are acquired. Those hallmarks include evading growth suppressors, sustaining proliferative signaling, avoiding immune destruction, deregulating cellular energetics, resisting cell death, enabling replicative immortality, acquiring genome instability, inducing tumor-promoting inflammation, inducing angiogenesis, and activating invasion and metastasis [104]. A better understanding of these processes and their regulation could potentially enable a more specific characterization of the epigenetic deregulation, and thereby allow clinicians to target more specifically processes that are modulated through the deregulation of epigenetic controls or by specific non-coding RNA networks. 

In summary, multiple alcohol effects on specific pathways relevant for carcinogenesis have been observed, both in human patients and in experimental animal studies. These studies do not exclusively fit into a model where one mechanism (acetaldehyde or oxidative stress) is responsible for all the effects of alcohol, or where one type of breast cancer would be particularly susceptible to alcohol use. However, to the extent acetaldehyde is the culprit (in specific circumstances), it generally requires high levels of alcohol use and/or a deficiency in the ability to metabolize acetaldehyde. To what extent acetaldehyde accumulation occurs in breast tissue may not be immediately obvious, since ethanol metabolism is predominantly hepatic. More indirect consequences of alcohol use, on estrogen levels, or the effects of estrogen, are another set of pathways by which alcohol could act more broadly, including in breast tissue. Thus, a broader perspective may be needed. Epigenetic mechanisms are likely to be affected through other mechanisms than oxidative ethanol metabolism and may be altered in multiple tissues. However, there is little specific understanding of the impact alcohol has on these regulatory mechanisms and, in particular there is no information on dose dependency and temporal consequences of these actions. Despite these insights, however, the overall lack of mechanistic understanding of these regulatory events is a major impediment in exploring causal factors that are relevant in human patients.

## 2. Alcohol Consumption and Breast Cancer: Epidemiological Studies

Chronic heavy alcohol consumption (drinking too much, too often) and binge drinking (drinking too much, too fast) are risky behaviors that could result in various pathological conditions, including cancer. Some epidemiological studies have suggested that even moderate alcohol consumption can increase the risk of breast cancer, although by a small extent [105]. Several recent articles found associations between modest or high alcohol consumption and a correspondingly moderate or higher increased risk of breast cancer and concluded that there is a positive dose-response relationship between alcohol drinking and breast cancer [106]. In addition, several articles reported an association between alcohol consumption and specific subtypes of breast cancer. However, alcohol was not associated with all breast cancer subtypes [107], illustrating once again how complex the relationship is between alcohol use and breast cancer, and how different modes of deregulating the underlying molecular mechanisms may play a role in breast cancer etiology. In Table 1, we summarize epidemiological studies published in the last two decades as an example of different studies with variable outcomes.

Examination of Table 1 reveals inconsistencies in the results. While some studies showed increased breast cancer risk from alcohol, others (e.g., studies 2, 6, 9, and 14) reported no association. It is important to point out that all observational studies were based on self-report, and there is a wide range of units of measurement of alcohol intake and the time periods in which these measurements were taken.

There seems to be quite a significant discordance between molecular and epidemiological data. For example, some molecular studies have ascribed the carcinogenic effect of ethanol, among other things, to estrogens, which is quite plausible since alcohol increases circulating estrogen levels in humans [124]. Most epidemiological studies show increased risk between alcohol consumption and the ER+ type of breast cancer. However, some epidemiological studies show increased risk in the ER− type (e.g., study 5 in Table 1); ER−/PR− (study 13); ER−/PR+ (study 13); and in TN tumors (studies 5, 7, 10, and 13). In addition, if estrogen is a factor, alcohol should increase the risk of both luminal A and luminal B tumors since both are ER+; however, increased risk was reported only in luminal A (study #4), indicating that other factors may mitigate or alter the susceptibility to estrogen. Furthermore, a 2017 study showed that consumption of 15 g of alcohol per day for 8 weeks by postmenopausal women had no effect on urinary estrogen metabolites [125].

Another area of discrepancy between epidemiology and molecular biology is the role of acetaldehyde. While acetaldehyde is postulated to play a role in breast cancer, three epidemiological studies have examined the role of ALDH2*2 (the defective enzyme that causes higher blood acetaldehyde levels in individuals who consume alcohol) in the development of breast cancer and reported no association between ALDH2*2 and risk of breast cancer [126]. Furthermore, two studies examined the association between ALDH2*2 and the risk of breast cancer stratified by alcohol consumption and concluded that there is no significant effect of the interaction between ALDH2*2 and alcohol consumption on the risk of breast cancer [127,128]. This prompted Playdon and colleagues in 2017 [129] to state that the mechanisms linking alcohol and breast cancer risk are incompletely understood. A recent study by Wang and colleagues [130], however, discussed several mechanisms by which alcohol may enhance the progression and aggressiveness of existing mammary tumors.

The central theme of observational epidemiology is studying conditions that are identified as being associated with one or more specific factors, and importantly, to assess likely causal relationships that could be used to inform health policy recommendations and suggest clinical interventions. When it comes to non-communicable diseases, major limitations of such studies include non-replicable and non-causal findings [131]. Indeed, numerous observational studies have reported substantial causal associations that could not be confirmed in large-scale randomized controlled trials (RCTs) [132,133,134,135,136]. Such spurious epidemiological findings are usually at least partly due to confounders that, by themselves, may not be causally related to disease but are associated with other variables that influence disease risk. Other factors include inevitable measurement errors in assessing both exposure to the particular condition of interest and to potential confounders [137,138]. These factors could be particularly relevant in the context of estimates of moderate alcohol consumption as obtained on the basis of self-reported consumption patterns (see below). A significant question in the field is, therefore, how investigators can enhance confidence in epidemiological findings and what can be done to increase validity of causal inferences. One traditional approach to answer such questions has been to combine the findings from multiple studies in a meta-analysis, as discussed below. 

## 3. Alcohol Consumption and Breast Cancer: Meta-Analyses

Several meta-analyses have examined the association between alcohol consumption and breast cancer risk (see Table 2). While some studies reported a weak, non-linear, positive association between alcohol consumption and breast cancer (study G), others (study F) observed a linear increase of breast cancer risk with an increasing level of alcohol consumption. It is worth noting, however, that study F defined moderate consumption as consuming ≤50 g per day, which is ~3.6 drinks; moderate consumption as defined by US dietary guidelines is up to one drink per day for women at 14 g per drink. Overall, studies agreed that increased breast cancer risk is associated with a high level of alcohol consumption, and the risk of breast cancer at a low level of alcohol consumption may require further investigation. Please note comments included in Table 2.

Meta-analyses collect and summarize information from many single observational studies which highlight improbably large effects despite weak evidence. The effect sizes usually shrink in meta-analyses, and some meta-analyses may control for potential confounding factors [148]. Nonetheless, even these analyses can be biased or misinterpreted [149,150].

There are numerous challenging issues concerning meta-analysis of observational epidemiological studies. These issues include: different study design (cohort, case-control, prospective, retrospective, etc.; vast variation in the quality of studies (sample size, drinking assessment, response rate, missing data, and choice of control in case-control studies, which are more prone to bias than cohort studies); variation in definition of various confounders, and in confounders included in various studies; selection bias in meta-analysis by including studies favoring certain outcomes, excluding non-English articles, or by abstracting incorrect data—the basic premise of meta-analysis being to average out errors, a slight change in selection criteria can get a different result; and under-reporting of alcohol consumed in observational studies. In fact, in a recent commentary, Ioannidis [151] echoed the limitations of meta-analyses in the field of nutritional epidemiology by stating that “meta-analyses become weighted averages of expert opinions.” In addition, it is well known that positive results are published, negative results are not. Sensitivity analysis should be used to demonstrate whether removing some of the selected studies alters the outcome. 

A 2017 meta-analysis [152] based on meta-analyses of 20 prospective studies reported a highly suggestive association between heavy alcohol intake and ER+ breast cancer [≥ 30 g per day of alcohol consumption versus nondrinkers, RR = 1.35 (1.23–1.48)]. The association between alcohol and ER-breast cancers was classified as weak, based on 17 meta-analyses. The study also reported a strong effect of “moderate” alcohol intake [12.5 g to 50 g per day—the equivalent of 1 to 3.7 drinks per day, versus non-drinkers, RR = 1.28 (1.10–1.49)]. It should be pointed out again that what is classified as *moderate* drinking is not moderate according to US dietary guidelines for alcohol consumption [153].

## 4. Association or Causation

Persistent methodological problems continue to plague the field of observational epidemiology, which may account for some of the inconsistency and uncertainty reflected in these studies. These include measurement error and confounding factors. Measurement error is especially problematic in alcohol research, which depends on self-report of alcohol consumption, an imperfect measure that more often than not leads to underestimates, particularly for moderate drinkers [154]. Additionally, measurement error has generated intense debate in, for example, the epidemiology of diet-induced cancer, leading to either missing important nutrition-cancer correlations, or reported false, deleterious, or protective associations [155]. Multiple confounders can collectively distort an exposure-outcome association and often yield misleading results [156]. These factors may invalidate causal interpretations of observed associations.

Numerous observational studies have associated alcohol consumption with breast cancer. While heavy alcohol consumption may increase the risk of breast cancer, linking alcohol consumption—especially in moderate amounts—to cancer, based solely on observational studies, is rarely definitive. It is intrinsically difficult to adequately account for confounding factors, including other lifestyle factors that are causally linked to carcinogenesis, and significant inaccuracies in estimates of long-term alcohol drinking history. The concept of causality is central to the development of interventions designed to reduce exposure to cancer-causing risk factors. Numerous causal risk factors have been linked to cancers (e.g., human papilloma virus and cervical cancer; ionizing radiation and leukemia; radon and lung cancer; aflatoxin and liver cancer; asbestos and mesothelioma—to name a few) only after the accumulation of sufficient evidence derived from epidemiological studies, clinical research, and mechanistic studies in laboratory animals [157]. The framework of causation in epidemiological studies was developed in 1965 by British medical statistician Sir Austin Bradford Hill [158], and includes the following minimal conditions needed to establish a causal relationship: temporality, strength of association, dose-response relationship, specificity, consistency (replicated results), plausibility (agrees with currently accepted understanding of pathological processes), experiment, coherence, and consideration of alternate explanations. Generally, epidemiological studies do not meet all of these criteria. The increasing availability of large scale molecular information science is entering a new era in which potential causal inferences are increasingly based on knowledge derived from mechanistic studies rather than exclusively relying on observational epidemiological studies, which by itself is generally “insufficient to establish causality” [157]. In fact, increasing concern about the validity of claims from biomedical research and particularly from observational research have been voiced [159,160]. Moreover, “media publicity about the findings is often based on novelty, rather than on reproducibility or scientific credibility.” [157]. Clearly, there is a need for independent supportive information to substantiate the observational associations. Different approaches are being developed that attempt to provide such independent validation.

### 4.1. Strengthening Causal Inference by Genetic Epidemiology through Mendelian Randomization

When confounding factors are present in a conventional epidemiological study but cannot be controlled for, Mendelian randomization is a widely used approach to address some of the problems of weak observational studies. Mendelian randomization combines genomic and epidemiologic methods to obtain more unbiased estimates of causal associations and offers a potential way to correct, in part, for the difficulties introduced by confounding factors and measurement errors.

A Mendelian randomization study published in 2007 by Smith and colleagues [161] using data from the British Women’s Heart and Health Study reported that many nongenetic modifiable risk factors occur in clusters. Thus, adjusting for confounders in observational studies will be hard because it will not always be clear which factors are confounders. The study concluded “These data illustrate why observational studies have produced misleading claims regarding potentially causal factors.” However, different approaches are being discussed on how to address these and other problems [162].

In alcohol research, no Mendelian randomization studies, to our knowledge, were performed that aimed to assess the relationship between alcohol use and breast cancer. However, alcohol-related studies using Mendelian randomization in other contexts have been based on the fact that genes involved in alcohol metabolism are polymorphic, and different allelic variants result in alcohol-metabolizing enzymes with various activities that can serve as proxies for different degrees of alcohol consumption. In other words, for polymorphic genes, allelic variants can produce physiological and/or biochemical states that encourage different levels of drinking and therefore reflect various levels of alcohol exposure at the population level. 

### 4.2. Marginal Structural Modeling and Agent-Based Modeling

Etiologies of complex diseases such as cancer are characterized by inference and multiple interacting causal effects [163]. Many statistical methods used in epidemiology include “main effects only,” regression-based models which assume that the actions of multiple causes are unidirectional and independent. To illuminate causality in epidemiological research, *marginal structural models* were introduced by VanderWeele et al. [164] in 2010. The advantage of using marginal structural models, as an alternative to regression models to test for sufficient cause interactions is that modeling assumptions are made on the relationship between the causes of interest and the confounding variables, and thus are more plausible. An alternative and complementary approach to elucidate complex causal interdependencies involves agent-based modeling, introduced in 2015 by Marshall and Galea [163]. Agent-based modeling is an apt way of tackling independence of causal effects and non-interference by simulating counterfactual outcomes. The goal is to make causal inferences as independent of theoretical opinions and expert judgement as possible [165]. To what extent these approaches could help support causal inferences from observational studies in particular on the impact of light or moderate alcohol consumption on breast cancer remains to be established.

### 4.3. Molecular Pathological Epidemiology

With the National Institutes of Health currently encouraging “precision medicine” to speed the movement of basic discoveries from the lab to the clinic [166], there are good reasons to consider applying approaches involving translational and interdisciplinary studies to the field of alcohol research. The concept of precision medicine implies that pathogenesis results from the unique interactions between cells involving genetic and epigenetic alterations, and lifestyle factors that vary between individuals.

The premise of classical epidemiology is that patients with the same diagnosis (e.g., breast cancer) have similar causes and disease progression despite variations in molecular pathology. Because each tumor possesses its own unique characteristics, such as molecular makeup, microenvironment, and communications within and between cancer and host cells, “it is essential that epidemiologic research rely on modern molecular classification of disease.” [167]. Therefore, inferences of causation can be strengthened by the interpretation of data from genomics, epigenomics, metabolomics, transcriptomics, exposomics, proteomics, molecular epidemiology, microbiome, and immunomics, among others [168]. Thus, causal claims should be based on different balances of contributions from empirical information (association studies) and mechanistic information [168].

**Figure 3:**
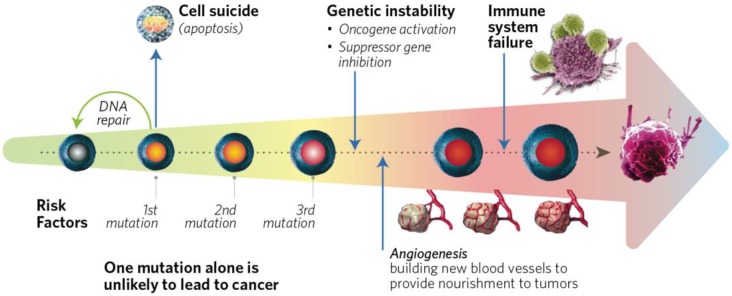
Cancer results from mutations involving genetic factors and epigenetic modifications affecting gene expression, a multitude of risk factors, lifestyle, microbiota resulting in vast biochemical changes that affect cell multiplication, survival, apoptosis, and evading surveillance by the immune system, culminating in cancer and metastasis.

Modern epidemiology is starting to incorporate genomic medicine and biomedical sciences, which is contributing a lot to the evolving field of molecular pathological epidemiology (MPE). The concept of MPE was developed in 2010 by Ogino and Stampfer [169]. These investigators noted that colorectal cancer, like breast cancer, is not a single disease, and yet was classified by epidemiological studies into a limited number of groups under the assumption that tumors with similar characteristics have arisen through common mechanisms. MPE integrates information about exposure, patient characteristics including immunity, and the consequent dysfunction of physiological events that lead to pathology (see Figure 3) [170]. 

For example, studies on colorectal cancer—a suitable disease model for the immunology-MPE approach, given rich microbiota and immune tissues of intestines, and the well-established carcinogenic role of intestinal inflammation—provided insights into immunomodulating effects of aspirin, vitamin D, inflammatory diets, and omega-3 polyunsaturated fatty acids [171]. Previously, the same authors found that higher pre-diagnosis levels of soluble tumor necrosis factor receptor type II (sTNF-RII) were associated with about a 48% increase in overall mortality in patients with colorectal cancer; however, among regular aspirin users, increasing levels of sTNF-RII were not associated with worse mortality [172]. Similarly, using MPE, Babic and colleagues [173] showed that higher levels of plasma leptin were associated with increased risk of pancreatic cancer in men, whereas in women a single nucleotide variant at the leptin receptor gene (rs10493380) was associated with pancreatic cancer risk. In addition, Yuan and colleagues [174] identified novel prognostic markers in patients with pancreatic cancer which implicated altered tricarboxylic acid metabolism in pancreatic cancer progression. They showed that two metabolites in the tricarboxylic acid cycle, isocitrate and aconitate, together with polymorphisms in Aconitase 1 were significantly associated with survival of patients with pancreatic cancer.

The transdisciplinary field of MPE “can advance biomedical and health research by linking exposure to molecular pathologic signatures, enhancing causal inference, and identifying potential biomarkers for clinical impact.” [167]. The field has so far mostly been focused on cancer, including, lung, pancreatic, and colorectal cancer. Characterization of molecular pathology in cancer is critical to link risk factors to plausible pathogenic mechanisms and cancer etiology [175], and consequently to predicting response or resistance to treatment.

MPE enables researchers to provide evidence about how endogenous and exogenous factors may interact and contribute to tumor evolution, and about the diversity of tumor phenotypes [176]. The concept that tumor subtypes that are genuinely biologically distinct are likely also to possess distinct etiologies has been well supported by the systematic analysis of etiologies and subtypes of bilateral breast cancers [177].

Molecular classification plays a pivotal role in MPE, with the underlying premise that breast cancers that share similar features are likely also to possess similar etiologies and behave in a similar manner in their progression and their response to treatment. Classically, etiologic heterogeneity of tumor subtypes is accomplished in case-control studies by comparing the odds ratios of candidate risk factors between the tumor subcategories and a common control group. This approach is incomplete, since many genetic risk factors for cancer have not been identified. Unfortunately, hitherto the MPE approach has not been used to evaluate the impact of alcohol use as a risk factor for breast cancer.

## 5. Conclusions

The discussion presented in this review has aimed to highlight both the broad scope of possible molecular mechanisms that may contribute to the onset or progression of cancers of the breast, as well as our ignorance of the detailed underlying processes and their regulation that dictates the outcome. Some of these potential processes influenced by alcohol use are likely to require relatively high levels of drinking and prolonged exposure. However, other mechanisms, such as those mediated by various epigenetic controls, are as yet poorly characterized and, as a result, remain unpredictable. The survey of the multiple epidemiological studies and meta-analyses also highlight the substantial variability that exists in the relative risk estimates, which are particularly difficult to interpret when light to moderate drinking frequencies are considered.

What is the reason to be so concerned about differentiating between potential cancer risk at high and moderate or low levels of drinking? Why is it important to have an understanding of the causative mechanisms of breast cancer and, more particularly, whether alcohol intake had anything to do with increasing the risk of specific subtypes of breast cancer? 

Alcohol has a social function that is important in many people’s lives. For the vast majority of drinkers this social function is associated with occasional or moderate drinking. The relative risk of negative consequences, including the relative risk of developing breast cancer, is therefore an issue that has to be weighed against these positives. Most observational epidemiological studies are in agreement that heavy drinking carries significant and diverse negative consequences and should be actively avoided. However, the negative consequences of light or moderate drinking are minimal, even in the majority of studies that show a detectable odds ratio for cancer or other diseases at these lower levels. 

The important question in this context is then whether a particular individual carries a higher risk than the general population as a consequence of specific susceptibilities that may vary from one person to another. It may be important, therefore, to be able to advise individual patients on specific vulnerabilities, either in their susceptibility to potential damaging effects of alcohol (e.g., those expressing the defective ALDH2*2 isoform) or with significant risk factors for developing breast cancer, BRCA1 or BRCA2 mutations the impact of which may increase their susceptibility to other potential risk factors. In addition, women with a positive family history of breast cancer, or are using contraceptive pills or hormone replacement therapy may be at higher risk.

A better understanding of the mechanistic basis of these susceptibilities then becomes a critical element. For this it is essential to obtain a more detailed knowledge of the molecular mechanisms by which alcohol use can impact cell function over prolonged periods. Such insights would enable the development of biomarkers that are mechanism-based and that, if well understood, could give guidance both to a physician who is asked to advise a patient, and to the individual patient who has to weigh these decisions. It would also facilitate the development of early detection tools that would help distinguish relatively innocuous indicators from more significant malignancies, and, of course, assist in the development of more individually targeted treatment modalities, the ultimate goal of “personalized medicine”. This is the promise of an approach such as that proposed under the umbrella of MPE. 

Breast cancer is a dreadful disease that can be treated effectively if caught early. In view of variable characteristics of different subtypes of breast cancer, the various susceptibilities of women to different risk factors and extents of exposure, as well as their various genetic susceptibilities, determining the molecular pathologic signatures is essential to facilitate better causal inference, and identify potential biomarkers for clinical impact and response to treatment. This endeavor will benefit greatly from the application of a multidisciplinary effort such as MPE, which encompasses various medical fields, including immunology, in addition to observational epidemiological studies. MPE facilitates the realization of precision medicine [171]. In the words of Nishi and colleagues [167]: “Molecular pathological characterization of diseases such as cancer is crucial to link risk factors to plausible pathogenic mechanisms, to estimate the natural history of an individual tumor, and to better predict the response/resistance to treatment or lifestyle intervention to maximize its benefit to each individual.”

## Figures and Tables

**Table 1:** Alcohol and Breast Cancer: Recent Epidemiological Studies.

Number	Study Design	Age (yearrs) at Baseline	Data Collection	Unit of Measurement	Outcomes	Ref
2017						
1	Longitudinal cohort NIH-AARP Diet and Health Study 190,325 postmenopausal women	55–70	Self-report	Avg. alcohol consumption (g/day) in 12 mo. before questionnaire completion	Similar associations were found between alcohol consumption (0.01–10 g/day) and ductal carcinoma in situ (HR = 1.05 [0.93–1.19]) on the one hand and invasive ductal carcinomas (HR = 1.03 [0.97–1.08]) on the other	[108]
2	Prospective cohort Nurses’ Health Study II 93,835 US women	27–44	Semi-quantitative food questionnaire	Calculated total daily alcohol consumption	Alcohol consumption was not associated with breast cancer risk overall for intake of ≥10 g/day vs. nondrinking (HR = 1.07 [0.94–1.22]) Positive association between alcohol consumption and breast cancer was found among women with a family history and folate intake < 400 μg/day (HR = 1.82 [1.06–3.12])	[109]
3	Sister Study 50,884 women	35–74	Self-report	Lifetime alcohol intake	High lifetime alcohol intake (≥230 drinks/year) increased breast cancer risk (HR = 1.35 [1.15–1.58]) For binge drinking (HR = 1.29, [1.15–1.45])	[110]
2016						
4	Prospective cohort Nurse’s Health Study 105,972 women	30–55	Semi-quantitative food frequency questionnaire	Cumulative average alcohol intake	10 g/day (HR = 1.1 [1.05–1.15 for luminal A]) and (HR = 1.16 [1.02–1.33 for HER2 BC]), but not with luminal B (HR = 1.08, [0.99–1.16)] Hormonal and non-hormonal mechanisms may play a role in this association	[111]
5	Case-control Carolina Breast Cancer Study; 781 Afr. Am. women; 1014 White women	25–50	Alcohol intake (self-report) most proximal to diagnosis	Drinks per week	Consuming more than 7 drinks/week was significantly associated with increased risk of ER− (OR = 2.17 [1.25–3.75]) and triple-negative (ER−/PR−/HER2–) in African American women but not in White women	[112]
2015						
6	Cohort study French E3N-EPIC 66,481 women	40–65	Self-report diet-history questionnaire	Cumulative average drinks/day	No association was found between drinking ≥2 drinks/day (beer, wine or spirits) and increase in breast cancer risk in premenopausal period ≥2 drinks/day of beer or wine was associated with increased breast cancer risk (HR = 1.85 [1.19–2.89], 1.33 [1.11–1.58]) in postmenopausal period This relationship was observed primarily in ER+/PR+ breast cancer risk (HR = 1.32 [1.08–1.60])	[113]
7	Prospective EPIC Study 334,850 women	35–70	Dietary and lifestyle questionnaires	Average lifetime alcohol intake	10 g/day increased breast cancer risk by 4.2% For intake of 5.1–15 g/day ER+/PR+ (HR = 1.09 [1.00–1.18]) ER+/PR− (HR = 1.13 [0.97–1.31) ER−/PR−/HER2– (HR = 1.18 [0.84–1.66]) Association between alcohol and breast cancer	[114]
8	Case-control 585 cases	28–90	Self-administered questionnaire	Total number of alcoholic drinks per week	<5 drinks per week was associated with increased risk of ER+ tumors In postmenopausal women: ER+ (OR = 2.32 [1.4–3.84]); ER+/PR− (OR = 2.92 [1.29–6.63]); ER+/PR+ (OR = 1.97 [1.08–3.57])	[115]
9	Prospective 45,233 women	30–49	Self-report	Current number of drinks/week converted to g/day	Alcohol intake was not statistically significantly associated with breast cancer risk, either overall or in different hormone receptor subtypes Overall, breast cancer risk increased with increasing alcohol intake among women with BMI <25 kg/m^2^	[116]
10	Prospective NHS	30–55	Self-report	Cumulative average intake per day	ER+/PR+/AR+ (HR per drink/day = 1.11 [1.06–1.17]) *(AR = androgen receptor)* ER−/PR−/AR− (HR per drink/day = 0.99 [0.88–1.12])	[117]
11	Prospective cohorts (2) Danish	50+	Self-report	Avg. drinks per week	Marked increase in breast cancer risk for hormone replacement therapy especially when combined with alcohol This effect was primarily restricted to ER+ cases	[118]
2013 and previous						
12	Case Control, 2013 Japanese cohort 1754 pre- and postmenopausal women	20–79	Self-reported alcohol drinking	Avg. consumption g/day	23 g/day (OR = 1.39 [95% CI: 1.07–1.80]) in postmenopausal women ER−/PR−/HER2+ (OR = 2.99 [1.08–8.26]) ER−/PR−/HER2− (OR = 3.72 [1.30–10.67]) No significant positive association was observed among premenopausal women Among postmenopausal women, no protective effect of folate was obvious across all subtypes, except ER−/PR−/HER2− (OR = 0.44 [0.20–0.96])	[119]
13	Prospective observational, 2011 Nurses’ Health Study 105,986 women	Avg. 60	Semiquantitative food frequency questionnaire	Avg. daily consumption in g/day	At 5–9.9 g/day, or 0.5–1.0 drink: ER+/PR+ (RR = 1.14 [1.02–1.28]) ER−/PR− (RR = 1.25 [1.01–1.54]) ER+/PR− (RR = 1.07 [0.85–1.34]) ER−/PR+ (RR = 1.47 [0.87–2.47]) Low levels of alcohol were associated with a small increase in breast cancer risk	[120]
14	Prospective control, 2011 Japanese cohort 19,227 patients	40–64	Food frequency questionnaire	Avg. consumption g/day	≥15 g/day, no significant relation to breast cancer risk Moderate drinking does not increase breast cancer risk	[121]
15	Prospective, 2010 50,757 pre- and postmenopausal Japanese women	40–69	Self-reported questionnaires	Average consumption g/week	>150 g/week RR = 1.78 (premenopausal), 1.21 (postmenopausal) No effect of folate, body weight, flushing due to defective ALDH2	[122]
16	Case control, 2008 437 women	25–85	Structured questionnaire administered by two interviewers	Average consumption g/day	<1.5 g/day decreases risk of breast cancer (OR = 0.58 [0.34–0.97]) Moderate drinking decreases risk of breast cancer	[123]

**Table 2:** Meta-Analysis Studies on Alcohol Consumption and Breast Cancer.

Study	# of Studies Included	Definitions of Drinking (g/day)	Relative Risk	Confidence Interval (95%)	Comments	Ref.
**A**	38 10 follow-up 28 case-control	13 g alcohol (~1 drink)/day	1.10	1.08–1.17	“Modest size of the association and variation in results across studies leaves the causal role of alcohol in question”	[139]
**B**	29 24 case-control 5 cohort	25 g/d (~1.8 drinks) 50 g/d (~3.6 drinks) 100 g/d (~7.1 drinks)	1.25 1.55 2.41	1.20–1.29 1.44–1.67 2.07–2.80	All doses are higher than moderate drinking	[140]
**C**	85 77 retrospective 8 prospective studies	Drinkers vs. non-drinkers Dose response	1.11	1.06–1.17 increased risk by 12% for 10 g/day	No quantification of amount of alcohol consumed	[141]
**D**	16 4 prospective cohort 12 case-control	Dose response for all ER+, ER−, PR+ & PR-tumors. Increased risk 10 g ethanol/day	12% ER+ 07% ER− 11% ER+/PR+ 15% ER+/PR− ER−/PR−	8%–15% 0%–14% 7%–14% 2%–30% No significant association	Two studies conducted in Asia and included in this analysis showed no association between alcohol and ER+ or ER− tumors Information on alcohol intake was collected after diagnosis	[142]
**E**	110 39 cohort 71 case-control		1.05	1.02–1.08	Heterogeneity across studies was high “Pool estimates should be interpreted with caution” Different drinking patterns were not taken into account	[143]
**F**	118 43 cohort 75 case-control	Light (≤12.5 g or ~1 drink) Moderate (≤50 g or ~3.6 drinks) Heavy (>50 g or >3.6 drinks)	1.04 1.23 1.61	1.01–1.07 1.19–1.28 1.33–1.94	According to US dietary guidelines moderate drinking is no more than one drink/day	[144]
**G**	16 13 case-control 3 cohort	Highest vs. lowest category of alcohol intake	1.28	1.07–1.52	Studies captured only ‘current’ drinking Weak nonlinear dose-response relationship Timing and quantification of alcohol consumption varied greatly	[145]
**H**	34 cohort studies	≤0.5 drink/day ≤1.0 drink/day	1.04 1.09	1.01–1.07 1.06–1.12	A small number of cohort studies in Asian populations were included	[146]
**I**	20 prospective cohort 1,089,273 women	≥30 g/day 5 to <15 g/day	1.35 ER+ 1.28 ER− BC even among women with high folate intake 1.12 ER+ 1.19 ER−	1.23–1.48 1.10–1.49 1.07–1.18 1.08–1.31	Alcohol was positively associated with risk of ER+ and ER− breast cancer Associations were similar beer, wine and spirits The associations with alcohol did not vary significantly by total folate intake	[147]
